# Risk of Permanent Corneal Injury in Microgravity: Spaceflight-Associated Hazards, Challenges to Vision Restoration, and Role of Biotechnology in Long-Term Planetary Missions

**DOI:** 10.3390/life15040602

**Published:** 2025-04-04

**Authors:** Jainam Shah, Joshua Ong, Ryung Lee, Alex Suh, Ethan Waisberg, C. Robert Gibson, John Berdahl, Thomas H. Mader

**Affiliations:** 1 Albert Einstein College of Medicine, Bronx, NY 10461, USA; 2 Department of Ophthalmology and Visual Sciences, University of Michigan Kellogg Eye Center, Ann Arbor, MI 48105, USA; 3 Houston Methodist Research Institute, Houston, TX 77030, USA; 4 Tulane University School of Medicine, New Orleans, LA 70112, USA; 5 Department of Clinical Neurosciences, University of Cambridge, Cambridge CB2 2PY, UK; 6 KBR, NASA Johnson Space Center, Houston, TX 77058, USA; 7 Vance Thompson Vision, Sioux Falls, SD 57105, USA; 8 NASA Ophthalmology Consultant, Moab, UT 84532, USA

**Keywords:** microgravity, corneal injury, ocular protection, bioprinting, corneal edema

## Abstract

Human space exploration presents an unparalleled opportunity to study life in extreme environments—but it also exposes astronauts to physiological stressors that jeopardize key systems like vision. Corneal health, essential for maintaining precise visual acuity, is threatened by microgravity-induced fluid shifts, cosmic radiation, and the confined nature of spacecraft living environments. These conditions elevate the risk of corneal abrasions, infections, and structural damage. In addition, Spaceflight-Associated Neuro-Ocular Syndrome (SANS)—while primarily affecting the posterior segment—has also been potentially linked to anterior segment alterations such as corneal edema and tear film instability. This review examines these ocular challenges and assesses current mitigation strategies. Traditional approaches, such as terrestrial eye banking and corneal transplantation, are impractical for spaceflight due to the limited viability of preserved tissues, surgical complexities, anesthetic risks, infection potential, and logistical constraints. The paper explores emerging technologies like 3D bioprinting and stem cell-based tissue engineering, which offer promising solutions by enabling the on-demand production of personalized corneal constructs. Complementary advancements, including adaptive protective eyewear, bioengineered tear substitutes, telemedicine, and AI-driven diagnostic tools, also show potential in autonomously managing ocular health during long-duration missions. By addressing the complex interplay of environmental stressors and biological vulnerabilities, these innovations not only safeguard astronaut vision and mission performance but also catalyze new pathways for regenerative medicine on Earth. The evolution of space-based ophthalmic care underscores the dual impact of space medicine investments across planetary exploration and terrestrial health systems.

## 1. Introduction

Human spaceflight offers an unparalleled opportunity to investigate the nature of life and biological systems in environments that cannot be replicated on Earth. Space-based research facilitates controlled studies in microgravity and radiation exposure, yielding insights into musculoskeletal degeneration, immune modulation, wound healing, and stem cell behavior—core concerns in space biology and space medicine [1,2,3]. These experiments are more than clinical curiosities; they inform new models of human physiology, offer therapeutic insights for Earth-based medicine, and contribute to a growing understanding of how life adapts to extreme environments [2].

These insights contribute to a broader scientific imperative: understanding life’s potential and persistence beyond Earth. Astrobiology—defined by the National Aeronautics and Space Administration (NASA) as the study of the origin, evolution, distribution, and future of life in the universe—sits at the intersection of biology, chemistry, planetary science, and astronomy [4]. As missions to Mars, Europa, and beyond seek biosignatures and signs of past or present habitability, astronauts serve as uniquely capable field scientists. Their adaptability, contextual reasoning, and ability to perform in situ analysis make human-led exploration essential to answering the foundational questions of astrobiology: *Where did we come from? Are we alone? Where are we going?* [4,5]. The success of this endeavor depends not only on engineering or propulsion—but on preserving human health, function, and perception in extreme environments.

As spaceflight transitions from short-duration missions in low Earth orbit to prolonged planetary exploration, the physiological challenges faced by astronauts will intensify [6]. Missions to Mars or deep-space outposts will expose crews to months or years of microgravity, heightened radiation, and limited medical support [7]. These conditions amplify the consequences of even minor physiological disruptions. In this context, safeguarding physiological and sensory systems is vital not only for performance, but for survival. Identifying and mitigating these biomedical risks is a prerequisite for enabling the next era of human exploration beyond Earth orbit.

Among these physiological systems, vision is paramount. Humanity’s pursuit of space exploration continually pushes the limits of human endurance, exposing astronauts to extreme conditions that challenge even the most resilient organ systems, including the eye. The cornea, which provides over two-thirds of the eye’s refractive power, is particularly vulnerable in microgravity, where precise vision is essential for mission success [8]. Even minor damage, such as a corneal abrasion from floating debris, can impact visual acuity and impair depth perception, jeopardizing vital tasks like extravehicular activities (EVAs). Similarly, visual distortions caused by stromal swelling or scarring can hinder key operations, from monitoring spacecraft systems to analyzing scientific data [9].

Ocular health in space is threatened by unique environmental stressors, including microgravity-induced fluid shifts, cosmic radiation, and confined living conditions, which increase the risk of corneal injury and visual impairment [8,9]. Spaceflight Associated Neuro-Ocular Syndrome (SANS), characterized by optic disc edema and posterior segment changes, has also been linked to fluid shifts that could potentially contribute to corneal edema and dry eye symptoms, representing a possible anterior segment vulnerability [9,10,11]. Looking beyond SANS, astronauts are also at increased risk for corneal anomalies to include abrasions, infections, and radiation-induced damage, emphasizing the urgent need for innovative protective strategies [10,12].

Traditional approaches, such as terrestrial eye banking and corneal transplantation, are unsuitable for future space missions to other planets due to the complexity of corneal surgery, anesthesia requirements, and the short viability of preserved corneal tissue, which lasts only 7–14 days [13,14]. These limitations underscore the necessity for advanced, sustainable solutions. One of the most promising innovations is 3D bioprinting, a groundbreaking technology capable of producing personalized, functional corneal constructs on demand. Although further research and refinement are clearly needed, this approach has the potential to revolutionize ocular healthcare during extended space missions [15,16].

This review examines the ocular challenges posed by spaceflight, evaluates current mitigation strategies, and explores the possible transformative potential of 3D bioprinting in corneal injury management. By addressing these vision-related challenges, this review outlines a path forward for protecting astronaut ocular health and catalyzing innovation in space-based healthcare.

## 2. Spaceflight Environment and Ocular Risks

### 2.1. Microgravity and Its Impact on Ocular Health

Microgravity profoundly affects human physiology, with significant implications for the visual and neurological systems. Cephalad fluid shifts induced by prolonged exposure disrupt normal pressure gradients, increasing intracranial pressure (ICP) and intraocular pressure (IOP), which directly impair ocular functions [17]. Elevated IOP has been associated with corneal edema and, in severe cases, bulla formation, further underscoring the vulnerability of the cornea in microgravity environments [18,19]. In this unique environment, astronauts face increased risks of corneal injuries such as abrasions and perforations, often caused by suspended particulates or high-velocity debris [8]. While these injuries are typically minor and treatable on the International Space Station, longer-duration missions may require advanced diagnostic and treatment strategies to ensure visual performance is maintained.

The cornea, a metabolically active tissue essential for maintaining transparency and refractive accuracy, is particularly vulnerable. Prolonged microgravity exposure has been linked to corneal edema, thickening, and tear film instability, which heighten the risk of abrasions and environmental damage [9,20,21]. These changes can degrade visual acuity and pose long-term risks to ocular health. 

**Figure 1:**
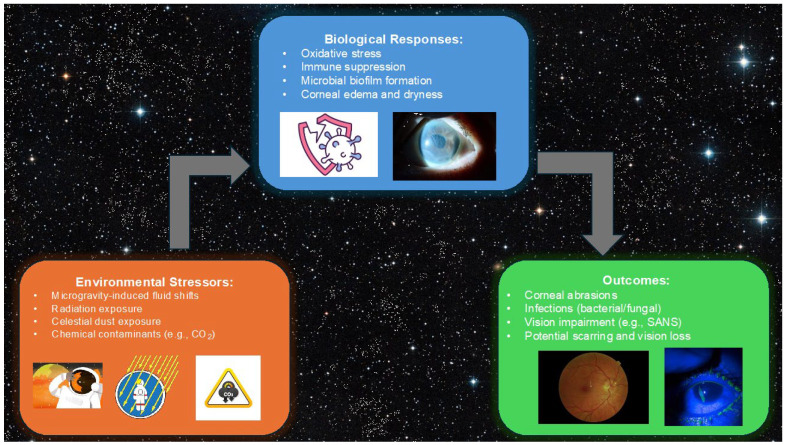
Environmental stressors in spaceflight, including microgravity, radiation, and celestial dust, lead to biological responses such as oxidative stress and immune suppression, resulting in outcomes like corneal abrasions, infections, and vision impairment.

Figure 1 illustrates the interplay between environmental stressors in spaceflight, such as microgravity-induced fluid shifts, radiation exposure, and celestial dust, and their downstream biological responses, including oxidative stress, immune suppression, and microbial biofilm formation. These factors collectively could potentially lead to adverse ocular outcomes, including corneal abrasions, infections, and potential vision loss [8,20,22].

Although SANS is primarily associated with optic nerve and retinal changes, fluid redistribution and increased intracranial pressure have been suggested as potential contributors to anterior segment alterations, including possible corneal edema and tear film instability [11].The proposed association between increased ICP and choroidal alterations highlights the multifactorial complexity of SANS [23].

Efforts to mitigate this microgravity-associated risks are gaining momentum. Gelatin methacryloyl (GelMA) hydrogels provide a biomimetic platform to study corneal biomechanics under pressure variations, offering insights into therapeutic approaches [24]. Similarly, computational fluid dynamics (CFD) models have deepened understanding of microgravity-induced hemodynamic changes, revealing increased shear stress in retinal and cerebrovascular networks [1].

As space exploration ventures further, addressing the ocular risks posed by microgravity remains vital. Developing innovative diagnostic and therapeutic strategies is essential to preserving astronaut vision and ensuring mission success in future space missions to other planets.

### 2.2. Corneal Bullae Observations in Mice

Animal studies also provide valuable insights into how microgravity impacts the cornea. The development of corneal bullae—fluid-filled blisters on the corneal surface—was observed in mice exposed to spaceflight conditions, highlighting the impact of microgravity on corneal integrity [21]. These bullae are believed to result from microgravity-induced oxidative stress and fluid shifts, which compromise corneal structure.

Although corneal bullae have not been reported in human astronauts, the potential for similar effects exists, particularly during extended missions. A novel 3D model using corneal keratocytes embedded in bioprinted matrices revealed cellular responses to microgravity-induced oxidative stress, mirroring observations of corneal bullae in mouse studies [25]. The impact on the cornea’s refractive properties underscores the importance of research into protective measures for ocular health during spaceflight [21,26].

### 2.3. Ocular Surface and Tear Film Changes

The tear film, essential for maintaining corneal hydration and protecting the ocular surface, is particularly susceptible to the effects of microgravity. In space, unpredictable pooling of tear fluid destabilizes the tear film, leading to symptoms of dry eye disease, a common complaint among astronauts [20,22]. Compounding these challenges are environmental factors such as low humidity aboard spacecraft, lower atmospheric pressure in space suits, and elevated CO_2_ levels, which accelerate tear evaporation and exacerbate irritation. Additionally, space radiation is a significant risk factor for dry eye disease in spaceflight. Several terrestrial studies corroborate the increased odds or incidence following radiotherapy for head and neck cancers [27]. Studies report a tolerance dose of 35–50 Gray for ocular surface changes (dry eye disease, tear break-up time) following radio therapy [28]. Space radiation is of lower magnitude with more unpredictable absorption rates. It is hypothesized that a greater risk for ocular surface disorder may occur in the spaceflight environment [29]. Furthermore, meibum can be modeled as a non-Newtonian fluid, and microgravity is known to decrease shear stress, theoretically leading to decreased meibum outflow [30,31]. Altered tear film and dry eye disease increase the risk of corneal abrasions, frequently reported during missions [20,32].

Tear film instability not only compromises the ocular surface but also heightens the risk of epithelial damage, abrasions, and ulcers. The cumulative effects of long-duration missions make effective hydration strategies a priority for preserving ocular health [33]. While artificial tears provide immediate relief, their transient effects highlight the need for more advanced interventions [13]. Recent advancements in bioengineered tear substitutes, including sustained-release formulations of anti-inflammatory peptides and biopolymers, have shown enhanced stability and efficacy in simulated microgravity environments. These innovations represent a promising approach to mitigating tear film instability during extended space missions [34].

### 2.4. Infections and Immune Suppression

Microbial keratitis represents a significant threat during spaceflight, exacerbated by a combination of immune suppression, microgravity conditions, and microbial contamination. Stress-induced immune dysfunction, driven by activation of the hypothalamic-pituitary-adrenal (HPA) axis and reduced cytotoxic T-cell activity, renders the ocular surface vulnerable to opportunistic pathogens [11,35]. Microgravity further destabilizes the tear film, weakening natural defenses and amplifying susceptibility to infections [22,36].

Microbial biofilms, inherently more resilient than planktonic bacteria, present a severe challenge in the confined spacecraft environment. Studies demonstrate that *Pseudomonas aeruginosa* and *Staphylococcus aureus* form denser biofilms under microgravity, exhibiting increased virulence and resistance to antimicrobials [37]. These adaptations, coupled with limited air circulation and sterilization challenges, significantly heighten the risk of ocular infections. Enhanced biofilm formation on spacecraft surfaces and water systems underscores the need for advanced countermeasures during long-duration missions [37].

Microgravity and associated spaceflight conditions can also alter the human microbiome, including that of the ocular surface. While direct studies on the tear film microbiome in microgravity are limited, terrestrial research highlights the importance of a balanced ocular microbiome in maintaining eye health [38]. Disruptions to this balance, potentially induced by spaceflight, could increase susceptibility to ocular infections [39]. Therefore, maintaining the stability of the ocular surface microbiome is crucial for astronaut health during long-duration missions.

Preventive measures, such as antimicrobial coatings on spacecraft surfaces and preemptive ocular defenses like antimicrobial contact lenses, are critical for managing these risks. Silver nanoparticle-infused materials and enhanced sterilization technologies have shown promise in reducing microbial adhesion and biofilm growth [37].

Herpes simplex virus (HSV) reactivation poses a significant concern during spaceflight. Stress-related immune suppression, isolation, and disrupted circadian rhythms can lead to reactivation of latent HSV-1, resulting in stromal keratitis characterized by inflammation, edema, and scarring, severely compromising visual acuity [11]. Prophylactic antiviral therapy, such as acyclovir or valacyclovir, is recommended primarily for astronauts with a history of recurrent HSV infections or those at high risk for reactivation [40] This targeted approach aims to minimize the risk of antiviral resistance associated with widespread prophylactic use [41].

### 2.5. Radiation Exposure and Corneal Damage

Galactic cosmic rays (GCR) and solar energetic particles (SEP) pose significant threats to ocular health during deep-space missions. These high-energy particles penetrate biological tissues, causing DNA damage, oxidative stress, and apoptosis in corneal and retinal cells. Such damage can lead to radiation keratitis and stromal scarring, resulting in permanent visual impairment [42]. Experimental evidence in mice shows that radiation exposure induces oxidative stress and apoptotic cell death, particularly under low-dose space radiation conditions, as observed in retinal endothelial cells, with potential implications for corneal health [43].

Radiation levels depend on mission distance from Earth and duration. For instance, astronauts on the International Space Station (ISS) are exposed to approximately 200 mSv annually, while missions beyond Earth’s magnetosphere, such as those to Mars, will expose astronauts to significantly higher doses. Prolonged exposure to GCRs and SEPs amplifies risks of cumulative tissue damage, including radiation-induced cataracts and retinal degeneration [44]. Galactic cosmic rays (GCR) consist mainly of protons and helium ions, with approximately 1% heavy ions. These heavy ions, known as high atomic number and energy ions (HZEs), include elements such as oxygen and iron, which generate dense ionization tracks that cause clusters of irreparable DNA damage [45].

To address these risks, current protective measures, such as polyethylene shielding, provide partial mitigation but remain inadequate against high-energy GCRs. Advanced materials like hydrogen-rich composites and novel protective visors are under development to enhance shielding efficacy for ocular tissues [44]. Additionally, biomimetic solutions, including GelMA hydrogel constructs, are being investigated for their resilience to radiation and potential as bioprinted corneal replacements. These constructs mimic the biomechanical and structural properties of the cornea while providing enhanced resistance to oxidative damage [24].

Further research into oxidative damage mechanisms and countermeasures is essential to safeguard astronaut vision during long-duration missions. Continued innovation in shielding technologies, coupled with regenerative medicine approaches, will be critical for ensuring mission success and preserving astronaut health.

## 3. Traumatic Corneal Injuries

### 3.1. Injury Mechanisms in Microgravity

Microgravity environments significantly elevate the risk of ocular injuries due to the unique dynamics of suspended particles. In the absence of gravity, debris remains airborne, increasing the likelihood of contact with the eyes. This risk is especially pronounced in spacecraft cabins, where floating particles and confined spaces exacerbate exposure. NASA mission records document over 70 cases of corneal abrasions, characterized as superficial corneal defects from foreign bodies or minor blunt trauma. This classification also includes conjunctival foreign bodies, conjunctival lacerations, and scleral injuries [22,46]. The microgravity environment exacerbates exposure risk, as suspended particles—including celestial dust, cargo debris, and suit-trapped contaminants—remain airborne, increasing the likelihood of ocular exposure and subsequent injury [22].

Beyond operational hazards, the impact of extraterrestrial materials, such as lunar or Martian dust, presents a unique challenge. These particles, often sharp and abrasive, can adhere to surfaces, including helmets and gloves, and subsequently cause severe corneal trauma [8]. The confined and cluttered nature of spacecraft further complicates the management of such risks, as floating particles are difficult to contain.

To mitigate these challenges, protective measures, such as advanced visor designs and improved air filtration systems, are essential. Furthermore, developing on-demand therapeutic solutions, including bioengineered tear substitutes and biocompatible wound dressings, could significantly reduce the impact of ocular trauma during missions [34]. These approaches underscore the importance of integrating injury prevention with innovative treatment strategies to safeguard astronaut vision.

### 3.2. Celestial Dust Hazards

Lunar and Martian dust particles present unique challenges to ocular health due to their sharp, abrasive characteristics, high adhesiveness, and reactive surfaces. Unlike terrestrial dust, which undergoes weathering processes, celestial dust and larger particles remain jagged and highly reactive because of their formation in low-gravity and vacuum conditions. These abrasive properties make them particularly harmful when they encounter human tissue, including the eyes [46,47].

During the Apollo missions, astronauts frequently reported ocular discomfort and irritation caused by lunar dust introduced into spacecraft after EVAs on the lunar surface. Efforts to brush or vacuum dust off spacesuits prior to cabin entry were largely ineffective, leading to widespread contamination within the cabin. This resulted in acute symptoms, such as watery eyes and erythema, which could impair vision and performance during critical mission tasks [48,49]. These experiences underscored the limitations of current protective measures and the need for improved protocols.

The small size of lunar dust particles, often less than 20 µm, increases the risk of inhalation and ocular exposure. This microscopic dust as well as larger particles not only cause mechanical abrasions due to their sharp edges but can also generate reactive oxygen species (ROS) when interacting with human tissue, contributing to oxidative stress and delayed epithelial healing. Prolonged exposure during long-duration missions could exacerbate these effects, leading to chronic damage to the cornea and other ocular structures [21,46,47].

To mitigate these risks, advanced protective measures are essential. High-efficiency particulate air (HEPA) filtration systems within spacecraft can significantly reduce airborne dust, while shielded goggles and improved spacesuit designs aim to minimize dust transfer into habitats [48,49]. Additionally, innovations such as bioengineered tear substitutes with anti-inflammatory properties and sustained-release formulations offer promising therapeutic options to manage irritation and enhance recovery following dust-related injuries [34].

Future planetary exploration missions must prioritize understanding the long-term impacts of celestial dust and larger particulate matter on ocular health. Research into its mechanical and chemical effects, coupled with the development of robust protective and therapeutic strategies, is critical to ensuring astronaut safety and the success of extended space missions.

### 3.3. Spaceflight Associated Neuro-Ocular Syndrome

SANS, along with its associated symptoms, was first described and formally identified in 2011 and has since become a critical health concern for astronauts on long-duration missions [50]. The syndrome is characterized by posterior segment changes, including optic disc edema, globe flattening, hyperopic shifts and choroidal folds, which impair visual function and highlight the vulnerability of ocular structures in microgravity [33,50]. These changes are primarily attributed to cephalad fluid shifts induced by microgravity, which increase intracranial pressure (ICP) and intraocular pressure (IOP), disrupting the delicate balance of cerebrospinal fluid (CSF) within the brain and the orbital optic nerve sheath [8,12,17].

SANS-related changes often develop gradually over the course of long-duration missions, with papilledema, optic disc edema, and choroidal thickening among the most common findings [50]. While early-stage papilledema is often asymptomatic, prolonged elevation of ICP can lead to irreversible visual field defects and optic atrophy [11]. Detection in space relies on optical coherence tomography (OCT), fundus photography, and ultrasound of the optic nerve sheath diameter (ONSD), all of which have been used on the ISS to monitor astronauts [11]. Prevention strategies focus on mitigating cephalad fluid shifts, including the use of lower-body negative pressure, head-elevated sleep postures, and pharmacological options such as acetazolamide, a carbonic anhydrase inhibitor that reduces ICP [33]. Early detection and intervention are crucial in preventing permanent vision impairment, underscoring the need for in-mission monitoring and countermeasures.

The functional consequences of SANS are significant. Astronauts frequently report blurred vision, which can compromise mission-critical tasks, such as spacecraft docking and EVAs [8]. These visual disturbances underscore the need for vision correction during space missions as well as comprehensive ocular monitoring during and after missions, covering both posterior and anterior segment changes [10].

Recent advancements in imaging technologies, such as optical coherence tomography (OCT), have been instrumental in detecting and tracking the subtle morphological changes associated with SANS. Tools like OCT allow for precise measurement of optic nerve head morphology, retinal thickness, and choroidal alterations, significantly improving our understanding of the syndrome’s pathophysiology [11,34]. However, the underlying mechanisms of SANS remain incompletely understood, necessitating further research into its multifactorial causes.

Preventive measures are essential to mitigate the risks posed by SANS. Countermeasures, such as lower body negative pressure devices, show promise in managing fluid shifts and reducing their ocular impact. Continued innovation in monitoring tools and countermeasures is vital to safeguarding astronaut health and ensuring the success of increasingly ambitious space missions [10,33].

### 3.4. Corneal Stromal Scarring Risk Assessment

Corneal stromal scarring, though extremely rare and not yet documented in astronauts, can have significant implications if it were to occur. Even minor visual impairments can hinder mission-critical tasks like navigation, system monitoring, and extravehicular activities. This risk arises from space-specific stressors, including microbial keratitis, herpes simplex virus (HSV) reactivation, and cumulative radiation exposure, which collectively create a high-risk environment for ocular damage during long-duration missions [22].

Microbial keratitis is a serious threat in microgravity, where immune suppression increases susceptibility to opportunistic infections. Stress-induced immune dysfunction and the unique microbial dynamics of spacecraft enhance the risk of infections progressing to scarring without prompt management [11,35]. Similarly, HSV reactivation, often triggered by elevated cortisol levels and immune suppression, leads to stromal keratitis characterized by inflammation, edema, and scarring, significantly impacting visual function [11,22].

Cumulative radiation exposure further compounds these risks. Galactic cosmic rays (GCR) and solar energetic particles (SEP) induce oxidative stress and apoptosis in corneal cells, as demonstrated in experimental models. Studies reveal that prolonged radiation exposure beyond Earth’s magnetosphere increases the likelihood of structural corneal damage, elevating visual risks for astronauts [36,42].

Mitigation strategies are critical for reducing these risks. Comprehensive microbial control measures, such as antimicrobial coatings and rigorous decontamination protocols, help prevent keratitis progression [35]. Antiviral therapies targeting HSV reactivation provide another layer of protection against vision-compromising infections [11,22]. Advanced shielding technologies, such as polyethylene alternatives and hydrogen-rich composites, minimize radiation exposure during deep-space missions [36,42]. Additionally, psychological interventions and circadian rhythm regulation address stress-induced immune dysfunction, further reducing ocular complications [11,35].

Innovative regenerative approaches offer long-term solutions for scarring. Bioengineered corneal replacements, such as those using transparent constructs made with UNION bioinks, replicate the cornea’s optical and biomechanical properties [51,52]. These constructs, incorporating astronaut-derived stem cells, demonstrate resilience to radiation-induced damage, offering a sustainable and personalized solution to address stromal scarring risks [25,53].

## 4. Current Management Strategies for Corneal Scarring

### 4.1. Standard Terrestrial Treatments

Corneal transplantation techniques have significantly evolved to address various extents of corneal damage. Among these, Deep Anterior Lamellar Keratoplasty (DALK) is a well-established approach for anterior corneal pathologies such as keratoconus. This technique spares the endothelium and Descemet’s membrane, thereby reducing graft rejection risk [44]. Enhanced precision through femtosecond laser and “big bubble” techniques has further refined outcomes, minimizing complications and improving long-term graft survival [54,55].

Penetrating Keratoplasty (PK) remains the gold standard for full-thickness corneal damage, offering robust solutions for severe scarring caused by conditions like herpetic keratitis [56]. However, PK involves complex surgical techniques, an operating microscope, the use of anesthetic, and is associated with higher risks of immunological rejection and prolonged recovery times, necessitating extended immunosuppressive regimens to mitigate complications [56,57]. Emerging innovations aim to address these challenges, including pre-surgical anti-inflammatory strategies to improve graft acceptance [57].

For endothelial dysfunction, Descemet’s Membrane Endothelial Keratoplasty (DMEK) and Descemet’s Stripping Automated Endothelial Keratoplasty (DSAEK) are commonly performed. While DMEK ensures faster visual recovery and lower rejection rates, its steep learning curve makes it less accessible in under-resourced settings [58,59]. DSAEK, though simpler to perform, has slower recovery rates but remains a viable alternative in resource-constrained environments [58].

Adjunctive therapies, such as anti-scarring agents and advanced ocular bandages, complement these surgical approaches. For example, molecular therapeutics like exosomes and growth factor inhibitors are under investigation for minimizing postoperative fibrosis and enhancing corneal clarity [60].

### 4.2. Limitations in Space

Adapting terrestrial transplantation techniques to space missions presents formidable challenges. Traditional eye banking is constrained by the short viability of donor tissues, typically limited to 14 days. While advancements like the Active Storage Machine extend tissue viability to three months, these remain experimental and unsuitable for long-duration missions [61]. Moreover, risks of microbial contamination during tissue storage and transport greatly detract from the feasibility of using donor corneas in microgravity environments [62].

In situ corneal transplants aboard spacecraft face significant additional obstacles. Maintaining sterile conditions during surgical procedures is nearly impossible due to the constraints of spacecraft design. The absence of sophisticated surgical tools, operating microscopes, and intensive postoperative care resources further limits the practicality of conventional transplantation in space [57,58]. Moreover, limited access to immunosuppressive medications during missions increases the risk of graft rejection, making traditional approaches less viable [58].

To address these barriers, bioengineered corneal substitutes and stem cell-based regenerative therapies are emerging as promising alternatives. These approaches leverage advancements in tissue engineering to produce biocompatible corneal constructs tailored to individual needs. For example, hydrogels derived from decellularized corneal extracellular matrix have shown potential in promoting scarless healing and could serve as off-the-shelf solutions for acute injuries in space [62]. Similarly, bioreactors capable of mimicking physiological cues and supporting extended tissue maturation are pivotal in overcoming the resource limitations of space exploration [53].

The integration of bioprinting technologies and compact bioreactors aboard spacecraft represents a critical innovation. These systems allow astronauts to produce corneal constructs on-demand using their own stem cells, mitigating the risks of immune rejection and reducing dependency on terrestrial resources. Furthermore, optimizing bioinks with enhanced viscoelastic properties ensures structural stability under microgravity conditions, paving the way for practical implementation during space missions [60,63].

By bridging terrestrial advancements with space-specific adaptations, these innovations hold the promise of revolutionizing ocular healthcare in space, addressing the unique demands of long-duration missions and habitation on other planets while advancing terrestrial ophthalmology practices.

## 5. Three-Dimensional Bioprinting of the Cornea

### 5.1. Advances in 3D Bioprinting Technology

**Figure 2:**
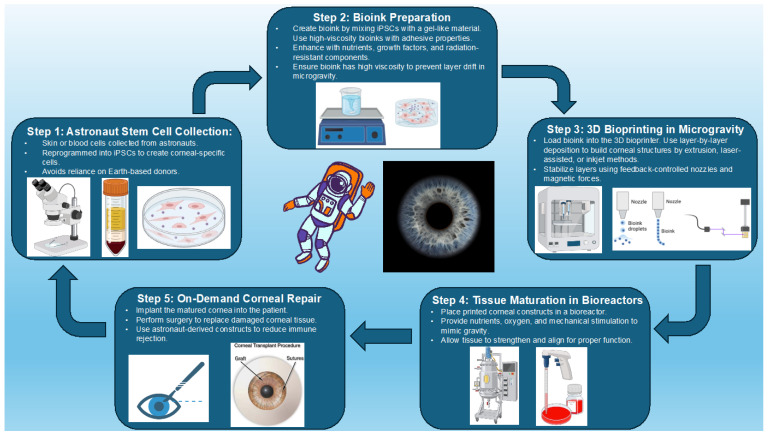
The 3D bioprinting pathway for corneal repair in space. This conceptual diagram illustrates the stepwise process, starting from astronaut-derived stem cell collection (Step 1) to the preparation of bioinks (Step 2), bioprinting in microgravity (Step 3), tissue maturation in bioreactors (Step 4), and culminating in on-demand corneal repair (Step 5). Each stage highlights the innovative integration of biotechnology to address the challenges of ocular injuries during spaceflight. Created with BioRender.com.

Three-dimensional (3D) bioprinting has revolutionized corneal regeneration by enabling precise deposition of cells and biomaterials to replicate the native cornea’s structure and function. Figure 2 illustrates a conceptualized stepwise process for 3D bioprinting of corneal constructs, integrating insights from recent advancements in stem cell integration, bioink preparation, and tissue maturation as described in the literature [15,26]. Among the leading techniques, extrusion-based bioprinting is widely used for its ability to handle highly viscous bioinks, which support high cell density and structural integrity. However, challenges in balancing cell viability with material stiffness remain, necessitating innovations in nozzle design and bioink formulations to optimize extrusion parameters, particularly under varying conditions such as microgravity [15,64]. These advancements are critical for improving the reproducibility and functionality of bioprinted corneal constructs [64].

Laser-assisted bioprinting offers unparalleled precision, making it ideal for creating intricate micro-architectures. This method enables the deposition of individual cells and bioinks, achieving excellent cell survival and precise stromal alignment [52,65]. However, its high cost and complexity limit scalability, highlighting the need for further technological improvements to enhance accessibility. Drop-on-demand bioprinting complements these approaches by offering precise droplet placement and uniform bioink distribution, supporting scalability while maintaining cellular organization [51,66].

Stereolithography-based techniques have made significant advances in producing mechanically stable and optically transparent corneal stromal constructs with precise collagen alignment. Incorporating bioactive molecules, such as growth factors and cytokines, promotes natural tissue healing and epithelial regeneration, enhancing the functionality of these constructs [67,68]. Hybrid scaffolds in multi-material bioprinting further improve structural fidelity and functionality, advancing the potential for clinical applications [69].

Microgravity environments provide unique advantages for 3D bioprinting, as the absence of gravity reduces sedimentation and deformation in bioinks, enabling the precise deposition of cells and materials. Techniques like extrusion-based and laser-assisted bioprinting perform particularly well in microgravity, facilitating the creation of intricate and functional constructs directly in space. These advancements reduce reliance on Earth-based resupply during long-term missions, addressing key logistical challenges [63,70].

Innovations in bioink formulations continue to expand the potential of 3D bioprinting. UNION bioinks, for example, offer tunable mechanical properties and high biocompatibility, supporting the creation of corneal constructs that replicate the layered architecture of the native tissue [51,52]. The incorporation of bioactive molecules, such as hyaluronic acid and VEGF inhibitors, enhances these bioinks by supporting cell differentiation and preventing neovascularization, which are critical for corneal clarity and function [68,71].

Despite these advancements, challenges remain in achieving long-term optical clarity, structural stability, and host tissue integration of bioprinted constructs. Ongoing refinements in bioprinting techniques, bioink formulations, and tissue maturation processes will be essential for transitioning this technology into clinical use [26,64]. An operating microscope, precise instruments, skilled surgical personnel, and the availability of appropriate anesthetic will complicate the use of this procedure. Also, follow-up of any surgical procedure will require the use of a slit lamp or similar optical device.

### 5.2. Stem Cell Integration and Tissue Engineering

The integration of stem cells in 3D bioprinting has driven significant progress in replicating the cornea’s complex structure and function. Corneal keratocytes encapsulated within collagen-based bioinks have shown high viability, contributing to the biomechanical stability, transparency, and refractive properties of bioprinted constructs. These advances underscore the importance of viable cellular components in mimicking native corneal tissue effectively [51,52].

Limbal stem cells (LSCs) play a pivotal role in regenerating the corneal epithelium, particularly under pathological conditions. Found at the limbus, LSCs are uniquely suited to repairing and maintaining the corneal surface. Techniques for ex vivo expansion of LSCs on biocompatible scaffolds, such as amniotic membranes or hydrogels, have demonstrated success in restoring epithelial integrity and reducing complications like neovascularization, making them essential for addressing limbal stem cell deficiency [72]. 

Embryonic stem cells (ESCs) and induced pluripotent stem cells (iPSCs) offer broader regenerative potential by differentiating into the primary cell types of the cornea—keratocytes, epithelial cells, and endothelial cells. Co-culture systems pairing these cells with engineered scaffolds or bioinks mimicking the extracellular matrix (ECM) provide necessary structural and biochemical support. However, challenges such as teratoma formation remain significant [73,74].

Advanced bioinks, like UNION bioinks, have further enhanced the mechanical properties and biocompatibility of bioprinted corneal constructs. These bioinks replicate the organized collagen fibrils within the corneal stroma, ensuring structural integrity and optical clarity. Embedding corneal epithelial cells within these bioinks has improved epithelial regeneration and cell viability, addressing challenges like maintaining epithelial integrity during extended space missions [25,51].

Biomimetic scaffolds have emerged as critical tools for creating environments conducive to cellular adhesion, proliferation, and differentiation. Hybrid scaffolds combining natural polymers, such as collagen and hyaluronic acid, with synthetic materials like polyethylene glycol (PEG), offer a balance of mechanical strength and transparency. These scaffolds facilitate the organized growth of stem cells into functional tissues, closely replicating the native corneal architecture [74,75].

Dynamic bioreactors have revolutionized tissue engineering by replicating physiological cues essential for in situ tissue maturation. These systems guide stem cell differentiation and extracellular matrix remodeling by simulating natural biomechanical environments. Advancements in microfluidic bioreactors, which replicate corneal biomechanical properties, have further improved the functional integration of bioprinted constructs, making them particularly valuable for long-duration space missions [51,75].

Despite these innovations, achieving fully functional, multi-layered corneal constructs remains challenging. Transparent replacements require precise alignment of stromal collagen and integration of functional epithelial and endothelial layers. Addressing issues like cell–biomaterial interactions, uniform stem cell distribution in bioinks, and potential immune responses are critical areas for continued research [26,65].

### 5.3. Addressing Space-Specific Challenges

As bioprinting technologies advance, their application in space exploration offers promising opportunities and significant challenges. Three-dimensional bioprinting offers a sustainable solution for corneal tissue generation during space missions, overcoming logistical limitations associated with transporting donor tissues. Unlike vascularized tissues, the cornea’s avascular nature simplifies bioprinting and makes it a prime candidate for space-based applications [15,65].

However, the microgravity environment introduces challenges. Bioink cohesion, layer stability, and structural integrity are all affected by the absence of gravitational forces, requiring adaptations to existing techniques and formulations. Feedback-controlled bioreactors have emerged as a key innovation, stabilizing layers and ensuring consistent cell viability during the bioprinting process [34,64]. These systems replicate physiological cues, such as oxygen gradients and mechanical forces, to enhance tissue maturation.

Sterility and precision are critical in space-based bioprinting. Enclosed sterile systems mitigate contamination risks, while modular bioprinters designed for space missions maximize resource efficiency. These compact units support multi-material capabilities, enabling astronauts to address a range of medical needs with limited onboard resources [52,69].

Innovative bioinks, such as those designed to remain stable under microgravity, address challenges related to layer cohesion and material flow. UNION bioinks, for example, provide the mechanical strength and biocompatibility necessary for creating corneal tissues in space environments [15,51]. Furthermore, incorporating autonomous diagnostic and therapeutic tools into bioprinting frameworks will expand the capabilities of astronauts to manage ocular emergencies, such as corneal ulcers or abrasions, without reliance on terrestrial support [63,64].

Future advancements in space bioprinting will focus on achieving long-term durability and scalability of constructs while integrating them with other space-based healthcare technologies. Addressing these challenges will be crucial for enabling sustainable medical support during deep-space missions and beyond.

### 5.4. Future Research Directions

The future of 3D bioprinting for corneal regeneration hinges on advancing bioreactor systems, optimizing bioink formulations, and personalizing treatment approaches. Bioreactors designed for in situ tissue maturation are central to enhancing the biomechanical stability and optical clarity of bioprinted corneas. These systems replicate the mechanical and biochemical cues essential for corneal development, simulating the natural biomechanical environment required for functional tissue maturation. This capability is especially valuable during long-duration space missions, where external resources are limited [26,51]. Dynamic perfusion bioreactors, providing continuous nutrient delivery and waste removal, show promise in improving tissue integration and functional stability [24].

Personalized medicine, particularly the use of astronaut-derived stem cells, represents a transformative direction in bioprinting research. Utilizing an astronaut’s stem cells minimizes immune rejection risks and allows for tailored therapeutic solutions. When combined with advanced bioinks, these stem cell-based approaches enhance the biological compatibility and functionality of bioprinted constructs. Bioinks enriched with growth factors, cytokines, or extracellular matrix components promote cell proliferation and differentiation, essential for constructing fully functional corneal layers [26,51].

Integrating bioprinting systems into space healthcare infrastructures will be pivotal for long-duration missions. Compact, modular bioprinters tailored to the constraints of space habitats could enable the on-demand production of corneal tissues and other critical medical constructs, reducing reliance on terrestrial resources [15]. Modular platforms that prioritize adaptability, precision, and resource efficiency are particularly suited to both terrestrial and extraterrestrial medical needs [65].

The unique demands of space exploration, including microgravity and resource limitations, present challenges that must be addressed to make bioprinting viable in extreme environments. Stable bioink formulations for microgravity conditions and sterilizable, reusable bioprinter components are essential for efficiency. Integrating bioprinting systems with AI-driven diagnostics and autonomous therapeutic modules could establish comprehensive frameworks for managing medical emergencies, such as corneal trauma or disease [34,63].

Future efforts should also focus on enhancing post-printing maturation processes. Dynamic bioreactors incorporating biomechanical stimulation, such as shear stress or compression, can accelerate tissue development while improving the structural and optical properties of bioprinted corneas. Advanced imaging tools for real-time monitoring will refine tissue development protocols, ensuring consistent quality and functionality [52,68].

By addressing these challenges, 3D bioprinting has the potential to revolutionize healthcare in space and on Earth. Continued innovation in bioreactor technology, stem cell integration, and bioprinter design will ensure the feasibility and effectiveness of this transformative approach, making it a cornerstone of modern medicine in extreme environments and beyond.

## 6. Strategic Planning and Risk Mitigation

### 6.1. Protective Eyewear

Adaptive visors are critical for mitigating two primary risks during space missions: high-energy radiation exposure and mechanical trauma from micro-debris. These visors incorporate advanced radiation-blocking coatings to shield ocular tissues from ultraviolet (UV) rays and galactic cosmic rays (GCRs), which accelerate conditions like keratitis and corneal scarring [76]. Nanomaterial coatings provide effective GCR protection while maintaining lightweight functionality for EVAs [25]. Impact-resistant materials, such as polycarbonate or advanced polymers, are integrated into visor designs to prevent corneal abrasions or punctures caused by micro-debris impacts [76].

Emerging technologies, like electrochromic visors, offer real-time adaptability to varying light conditions. By incorporating liquid crystal or photochromic materials, these visors automatically modulate opacity based on ambient light intensity, reducing glare and eye strain during transitions between light and shadow [32]. Active cooling systems further enhance visibility by preventing fogging in enclosed helmets, ensuring optimal performance during critical tasks.

Visors for deep-space missions also incorporate layered composite materials to disperse the energy of high-velocity micro-meteoroid impacts, enhancing durability. Self-healing materials, currently under investigation, could autonomously repair small abrasions, extending the lifespan and reliability of protective visors [25].

Radiation shielding continues to advance, with innovations focusing on hydrogen-rich materials and magnetic shielding systems. These materials provide superior attenuation of high-energy particles while maintaining visor structural integrity. For long-duration missions, visors integrated with dosimeters offer real-time radiation exposure monitoring, enabling astronauts to respond promptly to elevated radiation levels [26,51].

Future developments aim to reduce visor weight further while enhancing durability and functionality. Customizable designs tailored to individual astronauts and mission-specific hazards promise a new generation of protective eyewear, ensuring both safety and performance during deep-space exploration.

### 6.2. Artificial Tear Solutions

The spacecraft environment, characterized by low humidity, altered airflow dynamics, and microgravity, exacerbates dry eye syndrome (DES), increasing the risk of micro-abrasions, inflammation, and secondary infections [32]. Artificial tear solutions with sustained-release formulations help maintain hydration and reduce application frequency, making them ideal for extended missions [32,77]. However, fluid dynamics in microgravity can compromise the efficacy of traditional artificial tears, necessitating innovative approaches to maintain tear film stability [20,36].

Administering eye drops in microgravity presents unique challenges due to the behavior of liquids in a weightless environment. Surface tension prevents fluid from forming discrete droplets, leading to difficulties in controlled application [11]. Traditional dispensers may result in overdosing or medication waste [11]. To address this, NASA has collaborated with companies like Nanodropper to develop micro-volume dispensers that regulate ophthalmic drug delivery in space [78]. Additionally, alternative approaches, such as gel-based artificial tears and pressurized dispensing systems, are under investigation to improve medication adherence during long-duration missions [79]. Understanding and optimizing these first-line treatment methods are essential before considering more advanced corneal interventions, such as bioengineered tear substitutes and regenerative therapies.

Recent advancements include gel-based artificial tears and bioengineered tear replacements designed to mimic the natural tear film. Formulations enriched with hyaluronic acid provide enhanced lubrication and protection. High molecular weight (HMW) hyaluronic acid, in particular, reduces oxidative stress, exhibits anti-inflammatory properties, and improves tear retention, making it highly effective for managing DES in extreme environments [80,81]. Lipid-based formulations address evaporative dry eye by stabilizing the lipid layer and reducing tear evaporation, crucial for mitigating microgravity-induced tear film instability [81].

Nanoparticle-based tear substitutes represent a promising frontier in DES management. These advanced systems prolong hydration and incorporate antimicrobial and anti-inflammatory agents, directly addressing the increased risks of infection and irritation in microgravity [34]. Complementary technologies, such as neurostimulation devices like intranasal stimulators, promote endogenous tear production and maintain tear film homeostasis without reliance on gravity-dependent application methods [82].

The integration of anti-inflammatory agents or antimicrobial peptides into artificial tear formulations further enhances their efficacy by targeting the underlying causes of ocular surface damage. These formulations also support wound healing, a critical feature given the heightened risk of corneal abrasions in particulate-laden environments like lunar or Martian missions [20].

While artificial tear solutions remain a cornerstone of ocular health management during space missions, their incorporation into comprehensive preventive and therapeutic frameworks—including protective eyewear, telemedicine systems, and AI-driven diagnostics—ensures a robust approach to mitigating ocular risks. Together, these innovations safeguard astronaut health and contribute to the success of missions in challenging extraterrestrial environments.

## 7. Telemedicine and AI Support Systems

### 7.1. Remote Medical Assistance

Telemedicine bridges the gap between spacecraft crews and Earth-based specialists, enabling support for ocular emergencies through high-resolution imaging tools, such as anterior segment cameras and fundus photography devices. These systems facilitate detailed evaluations that can be transmitted for expert consultation, particularly for diagnosing conditions like corneal abrasions, foreign bodies, or retinal changes [77,83]. However, as missions extend into deep space, communication delays of up to 20 min necessitate onboard diagnostic autonomy and advanced remote healthcare solutions [84].

NASA’s early applications of telemedicine during the Apollo and Skylab missions integrated telemetry, biosensor harnesses, and remote imaging to monitor astronaut health in real time. These technologies have evolved into modern tools like the Bio-Monitor and Bio-Analyzer, which provide near-instantaneous diagnostic data onboard spacecraft [85,86]. In ocular health, onboard diagnostic systems combined with AI and telemedicine frameworks allow astronauts to perform initial assessments and interventions autonomously when Earth-based consultations are delayed.

Multifunctional modular imaging systems, such as point-of-care OCT and ultrasound devices, enhance diagnostic accuracy while optimizing resource allocation. These tools are critical for addressing emergencies like retinal detachments or corneal abrasions, which require immediate intervention to prevent vision loss [86,87]. The diagnosis and treatment of corneal abrasions would be greatly augmented using such imaging systems. Although retinal detachments could be diagnosed, the treatment options during space missions would be very limited. AI-enabled decision support further empowers astronauts by providing tailored treatment pathways, reducing dependence on Earth-based expertise, and ensuring timely management of ocular emergencies [84,88].

Advanced telecommunication systems, including satellite networks like the Indian Data Relay Satellite System (IDRSS), enhance contact between astronauts and ground-based medical teams. These systems enable real-time consultations and data transmission, significantly improving emergency care coordination [88]. Virtual medical avatars—a novel telemedicine concept—combine biosensor data with AI-driven analytics to create interactive diagnostic and decision-support tools for autonomous health management in deep space [87].

By integrating telemedicine with autonomous diagnostic and therapeutic systems, future missions can achieve a new standard of health management for deep-space exploration. These innovations equip astronauts to address ocular emergencies and broader health challenges, enhancing the safety and success of long-duration missions while advancing remote healthcare technologies for terrestrial applications.

### 7.2. AI-Driven Decision Support

AI-driven tools enhance autonomous ocular diagnostics by detecting conditions such as optic nerve edema, corneal ulcers, and retinal detachment. Machine learning models help astronauts prioritize treatments, ensuring timely interventions without Earth-based support [10,32,34].

For managing spaceflight-associated neuro-ocular syndrome (SANS), these technologies are indispensable. Continuous monitoring of metrics like intraocular pressure and optic disc morphology enables early detection of risks such as tear film instability or optic nerve changes before they become severe. Non-invasive imaging tools, including optical coherence tomography (OCT) and ultrasound, integrated with automated systems, enhance the proactive management of ocular complications [46,89].

In addition to diagnostics, AI-driven tools excel in providing actionable recommendations. For example, convolutional neural networks (CNNs) have been applied to optical coherence tomography (OCT) and fundus photography to detect and classify retinal and optic nerve conditions with high accuracy. These AI-assisted imaging techniques are particularly valuable for deep-space missions where communication delays of up to 21 min limit Earth-based consultation [81,82].

These systems are also critical for autonomous medical interventions. Predictive models can identify patterns in ocular health metrics to suggest countermeasures, such as targeted exercises or pharmacological treatments, mitigating SANS progression [90]. They facilitate remote surgical guidance and robotic interventions, addressing emergencies like corneal abrasions or foreign body removal without requiring onboard specialists [89]. By enhancing tele-ultrasound capabilities, AI tools guide astronauts in capturing optimal diagnostic images and interpreting them onboard, bridging gaps created by delayed communication with Earth [91].

Beyond real-time care, these platforms offer cognitive support during high-stress scenarios. Decision-making tools tailored to resource-limited environments ensure astronauts can independently manage ocular emergencies, even in the isolating conditions of deep space [91,92]. Future advancements, such as generative adversarial networks (GANs), promise to simulate rare conditions, enhancing diagnostic accuracy and model adaptability. Additionally, AI-driven self-experimentation frameworks could accelerate research into corneal adaptation to microgravity, bioprinted tissue viability, and fluid dynamics affecting tear film stability. These systems could autonomously analyze in-flight data, optimizing diagnostic and treatment protocols for space-based ocular health [93,94].

Beyond diagnostic applications, AI-driven chatbots could serve as auxiliary tools for astronauts by providing real-time guidance on technical troubleshooting, medical protocols, and translation support for international crews. These models can process extensive medical databases, offering decision support during emergencies or research activities where Earth-based consultation is delayed. However, the reliability of AI-generated outputs can vary, and there is a risk of generating inaccurate or misleading information. Therefore, rigorous validation and testing are essential before integrating these systems into critical mission operations [95].

By seamlessly integrating diagnostics, predictive analytics, and autonomous interventions, AI platforms empower crews to manage ocular health effectively. These advancements reduce reliance on Earth-based support, paving the way for enhanced medical autonomy during long-duration space exploration.

### 7.3. Contingency Plans for Planetary Missions

Medical kits for long-duration space missions must incorporate advanced tools, including portable 3D bioprinters for on-demand fabrication of corneal constructs and other tissues. Paired with reservoirs of stem cells and bioinks tailored for corneal repair, these bioprinters address severe injuries, such as ulcers or abrasions, in isolated environments [15,26].

Compact imaging systems, when integrated with therapeutic devices such as femtosecond lasers, can significantly enhance astronauts’ ability to autonomously manage acute ocular conditions. Femtosecond lasers have revolutionized ophthalmic surgeries, including corneal procedures and cataract surgeries, due to their precision and minimal tissue damage [22,76,96]. Automated diagnostic systems linked to AI platforms further streamline assessment and treatment, ensuring effective medical responses even during emergencies [97]. These systems provide real-time contextual recommendations, enabling astronauts to handle both acute and chronic conditions far from Earth [98].

The modularity of medical kits allows customization based on mission specifics, such as crew size, duration, and anticipated health risks. Standardized checklists and high-fidelity training simulations ensure astronauts can effectively utilize medical kits under high-stress conditions. Given the constraints of space missions, these kits must be designed to be compact, accessible, and multifunctional, ensuring that astronauts have the necessary tools to manage ocular injuries, including corneal abrasions and infections, with minimal external support [97].

Additionally, advanced countermeasures for risks associated with prolonged microgravity, such as cephalad fluid shifts leading to neuro-ocular complications, are essential. Equipment for intraocular pressure monitoring and interventions for managing spaceflight-associated neuro-ocular syndrome (SANS) ensure comprehensive ocular care [9].

By integrating cutting-edge diagnostic, therapeutic, and training resources, these medical kits optimize crew health and safety, enabling mission success despite the challenges of long-duration space exploration.

### 7.4. Astronaut Training Programs

Comprehensive astronaut training programs are essential for equipping crews to manage ocular health autonomously during space missions. Virtual reality (VR) simulations provide an immersive platform for practicing complex procedures, such as bioprinting and emergency ocular surgery, under microgravity conditions [10,32]. These simulations replicate real-time scenarios, including the assembly and operation of bioprinting systems to address corneal emergencies [53]. Training programs should also emphasize diagnostic techniques, enabling astronauts to identify retinal or corneal abnormalities and make informed decisions in resource-limited environments. Incorporating AI-driven decision support ensures astronauts are familiar with the tools they will rely on during missions [34,77].

To bridge the knowledge gap in applying terrestrial surgical techniques in space, astronauts may undergo VR-based procedural training before launch, simulating microgravity conditions for corneal imaging, bioprinting, and emergency interventions [99,100]. In-flight, AI-driven decision support systems provide real-time procedural guidance, enabling astronauts to autonomously manage ocular injuries with stepwise instructions [101]. Additionally, telemedicine-assisted procedures allow specialists on Earth to guide astronauts remotely using compact ophthalmic toolkits and imaging systems [22,83,84]. These advancements ensure that even non-medical crew members can perform critical interventions with minimal terrestrial dependence.

Virtual Reality (VR) simulations are valuable in ophthalmology for surgical training and can help astronauts practice managing ocular injuries in space. These simulations provide hands-on training for conditions like corneal abrasions and foreign body removal, ensuring astronauts can respond effectively to vision-threatening emergencies during long-duration missions [100]. Gamification and real-time biometric feedback enhance cognitive adaptability and motivation, crucial for coping with the isolation and stress of space missions [99]. Additionally, integrating diagnostic tools like SANS-CNN during training prepares astronauts to independently manage neuro-ocular conditions, addressing communication delays with Earth-based experts [101].

Strategic planning for planetary missions must integrate advanced medical technologies and astronaut training to address ocular health challenges in space. VR and AI-driven tools enhance procedural preparedness, ensuring astronauts can manage ocular conditions autonomously.

## 8. Conclusions

In summary, safeguarding corneal health during future space missions is critical for astronaut safety and mission success. The impact of ocular threats, such as trauma, radiation exposure, and dry eye syndrome, are amplified by the unique environment of spaceflight. Traditional solutions to corneal opacification like corneal transplantation and eye banking remain limited by reliance on Earth-based resources and logistical constraints. However, advances in 3D bioprinting, stem cell integration, and customizable bioinks offer transformative alternatives, enabling on-demand tissue fabrication and reducing dependency on terrestrial support [15,26]. While the use of 3D bioprinting for replacing opacified cornea during spaceflight or habitation on other planets is a promising long-range goal, there are currently numerous technical constraints that would be prohibitive. These include the need for sophisticated OR facilities and skilled surgical personnel in these environments.

Furthermore, complementary innovations such as adaptive protective eyewear, telemedicine systems, and AI-driven diagnostic tools are paving the way for safer and more autonomous healthcare solutions. These technologies not only mitigate immediate ocular risks but also showcase the broader potential of space-inspired advancements for improving healthcare on Earth [10,32].

As space missions grow longer and farther-reaching—extending toward Mars and other planetary bodies—ensuring sustained visual performance becomes more than a clinical challenge; it becomes an enabler of scientific exploration, planetary research, and the human presence beyond Earth.

Looking ahead, sustained interdisciplinary collaboration among engineers, clinicians, and researchers is essential to refine bioprinting systems, AI diagnostics, and telemedicine frameworks tailored for the space environment. Compact bioprinting units and modular healthcare technologies will play a pivotal role in overcoming the challenges of long-duration missions.

By prioritizing innovation in ocular healthcare, we not only protect astronauts during exploratory missions but also unlock new possibilities for advancing medicine in extreme and isolated environments. This dual benefit underscores the profound impact of space-driven innovations on both space exploration and Earth-based healthcare—and reinforces the broader scientific value of investing in life science research beyond our planet.

## Data Availability

Not applicable.

## Abbreviations

CFD: Computational Fluid Dynamics; CSF: Cerebrospinal Fluid; DALK: Deep Anterior Lamellar Keratoplasty; DES: Dry Eye Syndrome; DMEK: Descemet’s Membrane Endothelial Keratoplasty; DSAEK: Descemet’s Stripping Automated Endothelial Keratoplasty; GCR: Galactic Cosmic Rays; GelMA: Gelatin Methacryloyl; HZEs: High Atomic Number and Energy Ions; ICP: Intracranial Pressure; IDRSS: Indian Data Relay Satellite System; IOP: Intraocular Pressure; ISS: International Space Station; LSCs: Limbal Stem Cells; OCT: Optical Coherence Tomography; PK: Penetrating Keratoplasty; ROS: Reactive Oxygen Species; SANS: Spaceflight Associated Neuro-Ocular Syndrome; SEP: Solar Energetic Particles; UNION: Universal Orthogonal Network.
